# Supplementary material for: Factors influencing vaccination coverage among children age 12–23 months in Afghanistan: Analysis of the 2015 Demographic and Health Survey
Source: PLoS One. 2020 Aug 7;15(8):e0236955. doi: 10.1371/journal.pone.0236955 (PMC7413477; doi:10.1371/journal.pone.0236955)
Supplement: S1 Checklist — (DOCX) [file pone.0236955.s001.docx]

STROBE Statement—checklist of items that should be included in reports of observational studies

|  | | Item No. | | Recommendation | Page  No. | Relevant text from manuscript |
| --- | --- | --- | --- | --- | --- | --- |
| **Title and abstract** | | 1 | | (*a*) Indicate the study’s design with a commonly used term in the title or the abstract | 1-2 | Title: Factors influencing vaccination coverage among children age 12–23 months in Afghanistan: Analysis of the 2015 Demographic and Health Survey |
|  |  |  |  | (*b*) Provide in the abstract an informative and balanced summary of what was done and what was found | 2 | Nationally representative data from the 2015 Afghanistan Demographic and Health Survey were used for this study. A sample of 5,708 children age 12-23 months with a vaccine card and immunization history was analyzed. Multinomial logistic regression was used to identify significant relationships between cofactors and vaccination status.  In the study, 51% the subjects were boys, 48% were born at home, and 76% were residents of rural areas. Background characteristics positively associated with vaccination status included delivery in a health facility(RRR=2.5, 95% CI=1.9-3.3), maternal age of 30-39 years (RRR=2.2, 95% CI=1.2-4.1), attending at least four visits for antenatal care(RRR=2.7, 95% CI=1.7-4.5), health facility visit in the past 12 months (RRR=1.9, 95% CI=1.4-2.5), paternal professional occupation (RRR=4.9, 95% CI=2.0-12.3), family with richer wealth index (RRR=2.4, 95% CI=1.4-4.1), and living in the northeast region (RRR=2.2, 95% CI=1.2-3.9)were positively associated with vaccination status. Living in the southern region (RRR=0.3, 95% CI=0.2-0.5) was negatively associated with vaccination status. |
| Introduction | | | | | |  |
| Background/rationale | | 2 | | Explain the scientific background and rationale for the investigation being reported | 3-5 | Levels of morbidity and mortality from vaccine-preventable diseases have decreased in recent years due to administration of childhood vaccinations [1]. Every year, vaccination effectively prevents about 2-3 million child deaths. Nonetheless it is also estimated that vaccine-preventable diseases are still responsible for 1.5 million deaths each year among children under age 5 [2]. Previous studies have demonstrated that vaccination has a positive impact on the control of communicable diseases and decreases the number of disability-adjusted life years (DALYs) rates [3-5]. A study in Iran found that the DALYs rates for measles were 86.1/100,000 in 1990 and decreased to 5.6/100,000 in 2010 [3]. A recent study by the United Nations Inter-agency Group for Child Mortality Estimation found considerable progress in child survival during last three decades. It also showed that the under-5 mortality rate has been reduced by 58% since 1990, while the number of under-5 deaths declined from 12.6 million in 1990 to 5.4 million in 2017 [6].  Global data on vaccination in 2017 show that nearly 123 million infants worldwide received the recommended three doses of DPT, which indicates the successful activities of the Expanded Program on Immunization (EPI) [7]. To cover the significant need for vaccines, the World Health Organization (WHO) and UNICEF announced the period from 2011-2020 as the Decade of Vaccine [8].  In Afghanistan, the EPI was initiated in 1978. It is one of the main sub-components of the Basic Package of Health Services (BPHS) under the main component of child health and immunization. The EPI services are provided from the Health Sub-Center (HSC) level to the Provincial Hospital (PH) level. Vaccination is provided at all public health facilities free of charge [9]. The number of health facilities that provide vaccination services has increased from 1,575 in 2015 to 2,926 in 2018 [1].  The EPI, under direction of the Ministry of Public Health, implements eight most common vaccines to prevent most vaccine-preventable diseases in the country. These vaccines include BCG, measles, Oral Polio Vaccine (OPV), and Pentavalent (Diphtheria, Pertussis, Tetanus, hepatitis B, and Hemophilus influenza type B). The current EPI schedule is BCG and OPV0 at birth, Penta-1 and OPV1 in the 6^th^ week, Penta-2 and OPV-2 in the 10^th^ week, Penta-3 and OPV-3 in the 14^th^ week, measles-1 at 9 months and measles-2 at 18 months. All the vaccines except for measles-2 should be completed by all children before 1 year of age [9].  Despite the improvements in vaccination services over the past 40 years and the increased number of health facilities that provide vaccination services, the vaccination coverage in Afghanistan has remained low due to security and other related problems [10]. A study by Farzard et al. that used the Afghanistan Health Survey dataset revealed that full vaccination coverage was only 39%. This low vaccination level among Afghan children is of great concern [11]. Outbreaks of vaccine-preventable diseases still have a seasonal pattern in Afghanistan. For instance, around 25,000 measles cases were reported during the winter in 2017 [1].  Child mortality is higher in Afghanistan compared with other countries [12]. Afghanistan has poor health indicators, based on data from 2015, and was reported as one of the most dangerous countries for children, with one of every 18 children dying before reaching their first year [13]. In 2017, the under-5 mortality rate in Afghanistan was 67.9 deaths per 1,000 live births [6]. Based on 2018 data, WHO indicates that only 65% of children received DPT3, which placed Afghanistan among the 10 countries with the lowest DPT coverage in the world [14, 15].  Understanding factors that influence vaccination coverage is important to increase the vaccination coverage rate. Numerous investigations have found that the factors influencing vaccination coverage among children include sex of child, place of birth, maternal and paternal education, maternal and paternal occupation, number of antenatal care (ANC) visits, household characteristics, and sociocultural factors [16-18]. |
| Objectives | | 3 | | State specific objectives, including any prespecified hypotheses | 5 | Thus, this study was aimed at identifying the factors associated with vaccination status among children age 12–23 months in Afghanistan. |
| Methods | | | | | |  |
| Study design | | 4 | | Present key elements of study design early in the paper | 5 | This study used nationally representative data from the 2015 Afghanistan Demographic and Health Survey (AfDHS). The AfDHS used a cross-sectional study design. The sample was collected based on a two-stage stratified cluster sampling method to cover the entire population of Afghanistan. |
| Setting | | 5 | | Describe the setting, locations, and relevant dates, including periods of recruitment, exposure, follow-up, and data collection | 5 | The sample was collected based on a two-stage stratified cluster sampling method to cover the entire population of Afghanistan. |
| Participants | | 6 | | 1. *Cohort study*—Give the eligibility criteria, and the sources and methods of selection of participants. Describe methods of follow-up   *Case-control study*—Give the eligibility criteria, and the sources and methods of case ascertainment and control selection. Give the rationale for the choice of cases and controls  *Cross-sectional study*—Give the eligibility criteria, and the sources and methods of selection of participants | 5 | A complete list of enumeration areas (EAs) was used as a sampling frame to cover the whole population; it was provided by the Central Statistics Organization (CSO) of Afghanistan. In the first stage of sampling, a total of 950 EAs were selected with probability proportional to size, 260 in urban areas and 690 in rural areas. In the second stage, 27 households were selected per cluster using systematic random sampling. The AfDHS was conducted from June 2015 to February 2016 and collected data from 29,461 ever-married women age 15-49 [19]. |
|  |  |  |  | (*b*)*Cohort study*—For matched studies, give matching criteria and number of exposed and unexposed  *Case-control study*—For matched studies, give matching criteria and the number of controls per case |  |  |
| Variables | | 7 | | Clearly define all outcomes, exposures, predictors, potential confounders, and effect modifiers. Give diagnostic criteria, if applicable | 6-7 | Dependent variable Vaccination status was categorized as: fully vaccinated, partially vaccinated, and non-vaccinated. Fully vaccinated status was considered as having received all recommended age-appropriate vaccines such as BCG, Pentavalent, OPV and measles-1; partially vaccinated status was defined as having received some but not all vaccines; and non-vaccinated status was defined as not having received any vaccines. Independent variables The independent variables were selected from the AfDHS dataset based on prior knowledge and published literature. These variables include sex of child, birth order, place of birth, maternal education, maternal age, number of ANC visits, health facility visit in the past 12 months, maternal occupation, maternal autonomy, paternal education, paternal occupation (included as a variable because in Afghanistan it has a tremendous effect on managing family-decision issues including child vaccination), household size (number of persons who live together in the same dwelling unit), household wealth quintile, exposure to mass media, place of residence, and geographic location.  Place of birth was categorized as: in a health facility, or at home. Maternal age was classified as: under age 20, 20-39, 30-39, and 40-49. ANC visit was categorized as: no ANC visit, 1-3 visits, and 4 or more visits. Maternal autonomy was defined in three categories: yes when the mother played a role in making decisions on family visits, large household purchases, and own health care; some when she played a role in some of these decisions; and no autonomy if she did not have a role in any of the decisions.  Exposure to mass media was classified as: yes when the family watched television, or listened to the radio, or read the newspaper at least once a week; and no when the family did not have such access to the media. Household size was defined based on the number of persons living in the household and categorized as: fewer than 5, 5-9, 10-14, and more than 14. Geographic region was classified into seven regions based on the EPI 2013 report—central, eastern, northern, northeast, southern, southeast, and western. |
| Data sources/measurement | | 8* | | For each variable of interest, give sources of data and details of methods of assessment (measurement). Describe comparability of assessment methods if there is more than one group |  | *NA* |
| Bias | | 9 | | Describe any efforts to address potential sources of bias |  | NA |
| Study size | | 10 | | Explain how the study size was arrived at | 5 | The AfDHS was conducted from June 2015 to February 2016 and collected data from 29,461 ever-married women age 15-49 |
| Quantitative variables | 11 | | Explain how quantitative variables were handled in the analyses. If applicable, describe which groupings were chosen and why | | 7-8 | The data were analyzed using statistical software STATA/SE version 15.0. The sociodemographic characteristics and general information were presented by frequency and percentage. Bivariate analysis was performed to assess the relationship between the independent variables and the dependent variable using the Chi-square test. A multinomial logistic regression model was used to determine the significance of the factors related to vaccination status after controlling for other covariates. The results were presented as adjusted relative risk ratio (RRR) with 95% confidence interval (CI). A p-value of less than 0.05 was considered as statistically significant. All the estimates were weighted to represent the population at the national level. The effect of complex multistage sampling design was considered in the analysis. Missing data were coded as missing and included in the analysis, but were not reported in the final table. |
| Statistical methods | 12 | | (*a*) Describe all statistical methods, including those used to control for confounding | | 8 | A multinomial logistic regression model was used to determine the significance of the factors related to vaccination status after controlling for other covariates. The results were presented as adjusted relative risk ratio (RRR) with 95% confidence interval (CI). A p-value of less than 0.05 was considered as statistically significant. All the estimates were weighted to represent the population at the national level. The effect of complex multistage sampling design was considered in the analysis. Missing data were coded as missing and included in the analysis, but were not reported in the final table. |
|  |  |  | (*b*) Describe any methods used to examine subgroups and interactions | | 8 | A multinomial logistic regression model was used to determine the significance of the factors related to vaccination status after controlling for other covariates. The results were presented as adjusted relative risk ratio (RRR) with 95% confidence interval (CI). A p-value of less than 0.05 was considered as statistically significant. All the estimates were weighted to represent the population at the national level. The effect of complex multistage sampling design was considered in the analysis. |
|  |  |  | (*c*) Explain how missing data were addressed | |  | NA |
|  |  |  | (*d*) *Cohort study*—If applicable, explain how loss to follow-up was addressed  *Case-control study*—If applicable, explain how matching of cases and controls was addressed  *Cross-sectional study*—If applicable, describe analytical methods taking account of sampling strategy | | 8 | All the estimates were weighted to represent the population at the national level. The effect of complex multistage sampling design was considered in the analysis. Missing data were coded as missing and included in the analysis, but were not reported in the final table. |
|  |  |  | (*e*) Describe any sensitivity analyses | |  |  |
| Results | | | | | | |
| Participants | 13* | | (a) Report numbers of individuals at each stage of study—eg numbers potentially eligible, examined for eligibility, confirmed eligible, included in the study, completing follow-up, and analysed | | 8 | The analysis included a total of 5,708 children age 12-23 months. |
|  |  |  | (b) Give reasons for non-participation at each stage | |  | NA |
|  |  |  | (c) Consider use of a flow diagram | |  |  |
| Descriptive data | 14* | | (a) Give characteristics of study participants (eg demographic, clinical, social) and information on exposures and potential confounders | | 9 | Table 1 |
|  |  |  | (b) Indicate number of participants with missing data for each variable of interest | | 9, 12, 14 | Table 1, 2 - 4 |
|  |  |  | (c) *Cohort study*—Summarise follow-up time (eg, average and total amount) | |  |  |
| Outcome data | 15* | | *Cohort study*—Report numbers of outcome events or summary measures over time | |  |  |
|  |  |  | *Case-control study—*Report numbers in each exposure category, or summary measures of exposure | |  |  |
|  |  |  | *Cross-sectional study—*Report numbers of outcome events or summary measures | | 11-14 | Table 2-4 |
| Main results | 16 | | (*a*) Give unadjusted estimates and, if applicable, confounder-adjusted estimates and their precision (eg, 95% confidence interval). Make clear which confounders were adjusted for and why they were included | | 11-14 | Table 2-4 |
|  |  |  | (*b*) Report category boundaries when continuous variables were categorized | |  |  |
|  |  |  | (*c*) If relevant, consider translating estimates of relative risk into absolute risk for a meaningful time period | | 12-13 | After controlling for other covariates, characteristics of children age 12-23 months most likely to be associated with a greater relative risk of full or partial vaccination, versus no vaccination, were: delivery in a health facility, maternal age, number of ANC visits, health facility visit in past 12 months, paternal occupation, household wealth quintile, and geographic region.  Children born in a health facility compared to those who were born at home had 2.1 times higher relative risk of being fully vaccinated compared to non-vaccinated (RRR=2.1, 95% CI=1.5-2.8), and 1.3 times higher relative risk of being partially vaccinated compared to non-vaccinated (RRR=1.3, 95% CI=1.0-1.7). Children of mothers age 30-39 at delivery compared to children of mothers under age 20 at delivery had 2 times higher relative risk of being fully vaccinated compared to non-vaccinated (RRR=2.0, 95% CI=1.0-4.0).  Children whose mothers made 1-3 ANC visits compared to no ANC visit had 70% higher relative risk of being fully vaccinated compared to non-vaccinated (RRR=1.7, 95% CI=1.3-2.3), and 60% higher relative risk of being partially vaccinated compared to non-vaccinated (RRR=1.6, 95% CI=1.2-2.1). Similarly, attending ANC at least four times compared to no ANC visit had 3.2 times higher relative risk of being fully vaccinated compared to non-vaccinated (RRR=3.2, 95% CI=1.9-5.3), and 2 times higher relative risk of being partially vaccinated compared to non-vaccinated (RRR=2.0, 95% CI=1.2-3.5). Children whose mothers visited a health facility in the past 12 months compared to those who did not have any visit had 90% higher relative risk of being fully vaccinated compared to non-vaccinated (RRR=1.9, 95% CI=1.4-2.5), and 70% higher relative risk of being partially vaccinated compared to non-vaccinated (RRR=1.7, 95% CI=1.2-2.2). |

Continued on next page

| Other analyses | 17 | Report other analyses done—eg analyses of subgroups and interactions, and sensitivity analyses |  | NA |
| --- | --- | --- | --- | --- |
| Discussion | | | | |
| Key results | 18 | Summarise key results with reference to study objectives | 19-20 | This study found that the overall coverage of full vaccination was low among children age 12-23 months in Afghanistan—highest in the central region and lowest in the southern region. Furthermore, this study identified some of the key factors associated with vaccination status such as; Children of mothers age 30-39 at delivery compared to children of mothers under age 20 at delivery had 2 times higher relative risk of being fully vaccinated compared to non-vaccinated. Children born in a health facility compared to those who were born at home had 2.1 times higher relative risk of being fully vaccinated compared to non-vaccinated. Children whose mothers made 1-3 ANC visits compared to no ANC visit had 1.7 times higher relative risk of being fully vaccinated compared to non-vaccinated. Children whose mothers visited a health facility in the past 12 months compared to those who did not have any visit had 1.9 times higher relative risk of being fully vaccinated compared to non-vaccinated. Children in households in the richer wealth quintile compared to the poorest had 2.6 times higher relative risk of being fully vaccinated compared to non-vaccinated. Children in the northeast region compared with central region had 3.2 times higher relative risk of receiving all basic vaccines. Children in the southern region, however, had 1.7 times lower risk of being fully vaccinated versus non-vaccinated than children in the central region. |
| Limitations | 19 | Discuss limitations of the study, taking into account sources of potential bias or imprecision. Discuss both direction and magnitude of any potential bias | 19 | Due to security problems in some parts of the country, the survey teams were not able to collect the relevant data in some areas. These areas were mainly in the rural districts of Uruzgan, Zabul, and Helmand provinces, which belong to the southern region of Afghanistan. The vaccination rates and influencing factors may be different in those areas not covered in the survey. |
| Interpretation | 20 | Give a cautious overall interpretation of results considering objectives, limitations, multiplicity of analyses, results from similar studies, and other relevant evidence | 18 | The main determinants for completeness of vaccination among children in the study were maternal age, delivery in a health facility, more ANC visits, health facility visit in the last year, paternal occupation, wealth quintile, and geographic region. |
| Generalisability | 21 | Discuss the generalisability (external validity) of the study results | 19 | The larger sample size of the DHS data allowed us to look for many associations |
| Other information | |  | | |
| Funding | 22 | Give the source of funding and the role of the funders for the present study and, if applicable, for the original study on which the present article is based |  | NA |

*Give information separately for cases and controls in case-control studies and, if applicable, for exposed and unexposed groups in cohort and cross-sectional studies.

**Note:** An Explanation and Elaboration article discusses each checklist item and gives methodological background and published examples of transparent reporting. The STROBE checklist is best used in conjunction with this article (freely available on the Web sites of PLoS Medicine at http://www.plosmedicine.org/, Annals of Internal Medicine at http://www.annals.org/, and Epidemiology at http://www.epidem.com/). Information on the STROBE Initiative is available at www.strobe-statement.org.
